# Goal Setting for Aging Adults and Care Partners: A Scoping Review

**DOI:** 10.1093/geroni/igad135

**Published:** 2023-12-22

**Authors:** Kate Perepezko, Pamela Toto, Mary Hitchcock, Beth Fields

**Affiliations:** National Center on Family Support, University of Pittsburgh, Pittsburgh, Pennsylvania, USA; Department of Occupational Therapy, University of Pittsburgh, Pittsburgh, Pennsylvania, USA; School of Nursing, University of Wisconsin-Madison, Madison, Wisconsin, USA; Department of Kinesiology, University of Wisconsin-Madison, Madison, Wisconsin, USA

**Keywords:** Assessment, Caregiving—informal, Function/mobility, Independence, Person-centered care, Physician–patient communication/relationships

## Abstract

**Background and Objectives:**

Evidence demonstrates that goal-setting and care partner support help aging adults improve their health. Less is known about how aging adults and care partners collaboratively participate in goal setting, revealing a potential gap in care delivery processes. The current review describes the scope of the literature on this topic.

**Research Design and Methods:**

A search was conducted in several relevant databases and 1,231 articles were screened for the following inclusion criteria: (a) participants included aging adults (50+ years) and care partners, (b) goal setting was conducted, and (c) articles were in English.

**Results:**

Common goals reported by aging adults were independence, improving or maintaining functioning, addressing symptoms, and remaining socially active. Care partners listed similar goals but also identified accessing services and supports as important. The level of care partner involvement varied across articles, with some care partners serving in a supportive role, some setting goals concurrently with the aging adult, and others setting goals on behalf of the aging adult.

**Discussion and Implications:**

This review revealed concordant and discordant prioritization of goals within dyads. These findings illustrate the importance and potential complexity of including care partners in the goal-setting process. We also found that collaborative goal-setting and care partner-directed goals are scarce, indicating the need for additional work in this area. Collaborative goal setting aligns with person and family-centered care approaches and can contribute to better care plans that meet the needs of aging adults and their care partners.

**Translational Significance:** Goal setting can facilitate person- and family-centered care; however, there is inconsistency in how care partners are included in goal setting. This review explores the literature on goal setting among aging adult and care partner dyads. Evidence suggests that dyads report concordant and discordant goals and reveal variability in the level of care partner involvement in goal setting. These findings highlight the need to develop methods to systematically involve care partners in goal setting. Findings also suggest that goal setting should be incorporated into preventative care plans to meet the priorities of aging adults and their care partners.

As our aging population continues to grow, it is becoming increasingly important that care planning practices are designed to meet the needs of aging adults (1). One method to provide this care is by employing person-centered approaches to increase the likelihood that care planning incorporates patient priorities and goals. These approaches can facilitate discussions between clinicians and patients so that patient choice is emphasized, shifting away from the traditional medical model approach (2). Person-centered approaches are particularly important for the aging population and people with chronic conditions due to the heterogeneity of these conditions and the lack of a gold standard treatment that is universally effective (3–6).

Person-centered care planning is facilitated by goal setting, an effective and frequently used method to improve health outcomes in a variety of settings and populations (7–9). Evidence suggests that incorporating goal-setting practices into regular clinical care can improve the relevance of therapeutic interviews, clarify treatment purposes with patients and their families, and improve patient satisfaction and quality of life (9–11). Goal setting has traditionally been employed in person-centered approaches to improve the alignment of a treatment plan with a person’s priorities and specific health conditions. However, these approaches are often flawed as they do not systematically reference the input of a key member of an aging adult’s care team, their care partner.

Care partners, people who provide unpaid care for their family or friends, are common, particularly for aging adults as they experience changes in sensory, cognitive, or physical functioning (12). Care partners frequently assist aging adults during medical visits and at home, support symptom management, increase treatment adherence, and coordinate services (13–16). However, while studies indicated that better coordination between clinicians and care partners results in better patient outcomes, including improved symptom management, functioning, and mental health (17,18), care partners are not always included in specific care planning activities, like goal setting (13–15). In fact, a recent scoping review by Raj et al. (2023) revealed that care partners are rarely included in the health care delivery process despite their critical role in care coordination. This stark omission in routine practice indicates that important information might be missing from care planning, as care partners can spend an average of 20 h per week providing care for an aging adult and therefore likely possess critical information about the aging adult’s functioning and well-being that can inform their care (15). To help address this gap in practice, systems and clinicians can strive to actively involve care partners in delivery processes by using family-centered approaches.

Family-centered approaches can promote a partnership between the patient, their family, and the clinician in care planning activities such as goal setting. In comparison to person-centered approaches, family-centered care emphasizes information sharing and participation among the entire team—the patient, their family, and the clinician (19). Although there is growing evidence to support family-centered approaches that incorporate both aging adult and care partner perspectives, these approaches are not universally or systematically employed (13). This is problematic as the recently released National Strategy to Support Family Caregivers (20) prioritizes care partner engagement. Therefore, it is timely to examine the scope of the literature related to goal setting among aging adults and their care partners. The current scoping review aimed to:

1. Describe the scope of the literature on the use of goal setting for aging adults and their care partners. This will include describing characteristics such as: the type of goals (eg, health management, behavior goals, etc.) reported by care partners and aging adults, disease/condition of aging adult, setting of study, how goals are reported (eg, self-report, questionnaire, and smart goal), and goal attainment.
2. Identify gaps in the literature on the use of goal setting for aging adults and their care partners to inform future research directions.

## Method

### Search Strategy

Searches of four relevant databases (PubMed, CINAHL, PsycINFO, and SocINDEX) were conducted in November 2022 to identify articles that examined goal setting for aging adults and their care partners. We did not limit our search to a specific time frame, searching databases of all records from their inception. Search terms were developed by a research librarian (M.H.) and modified by the team until all were satisfied with initial search results and exemplar articles were located within the test results. All searches were performed using terms in English. The search strategy for each database is shown in Supplementary Material, Section 1.

### Study Selection

Studies were included in the review if they met the following criteria: (a) published in English; (b) study participants include both patients classified as aging adults (50 years and older) and care partners (age 18 years and over) who provide unpaid care or support for an aging adult (eg, family, friend, neighbor, and nonrelative); (c) goal-setting was mentioned in the methods section as part of the study; and (d) study participants reported a goal. Studies were excluded from the review if they met the following criteria: (a) article not available in English; (b) article is a review article (eg, narrative review, systematic review, and scoping review); and (c) article is grey literature (dissertations, op eds, etc.). We did not limit our search to any specific geographic area.

**Figure 1.**
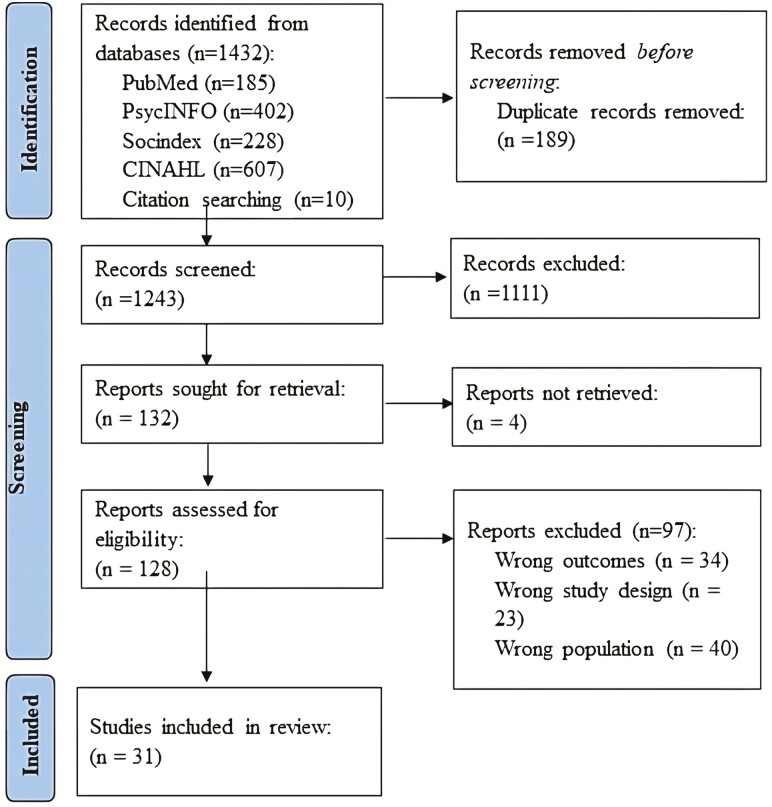
PRISMA flow diagram. (21)

Figure 1 shows the selection process for included articles. Articles were received from each database and imported into Covidence, removing any duplicates. Next, four reviewers (T.A., K.V., C.S., and B.K.) screened the titles and abstracts of articles based on the inclusion and exclusion criteria. Disagreements during this stage were resolved by another reviewer (K.P.). Then, the full-text articles were screened for inclusion criteria. Again, any discrepancies were adjudicated by an independent reviewer (B.F.). The reference lists of the remaining 29 articles were scanned to identify other possible studies that may have been missing from the original search. This scan yielded 10 additional published articles. After screening those articles, two met the inclusion criteria and were included in the review. A total of 31 articles, herein referred to as studies, were selected for this review.

### Data Extraction and Synthesis

A full-text screening questionnaire was developed by K.P. and B.F. to capture relevant data from included studies (Supplementary Material, Section 2). Major extraction categories included: general study characteristics, characteristics of the aging adult, characteristics of the care partner, goal-setting content, and other notes/relevant information (eg, limitations listed, author notes on interpretation). Two reviewers (K.P. and S.A.) extracted data from the studies that made it through to the full-text screening stage. Disagreements were discussed in periodic team meetings and resolved through discussion.

After extraction was completed a descriptive analysis (frequencies and means) of relevant data was summarized. The remaining data from the extraction was reviewed for themes using an inductive thematic analysis approach (22). Consistent with scoping review approaches, we did not conduct a quality assessment of included studies in this review (23).

## Results

### Participant and Study Characteristics

We identified 31 studies to include in our review. These studies were published between 1998 and 2022. Methodologies employed in studies varied, with some authors using qualitative methods (*n* = 14), others using quantitative methods (*n* = 11), and some using a mixed methods approach (*n* = 6) to investigate goal setting. Most study designs were observational (*n* = 24), some were interventional (*n* = 6), and two studies were longitudinal. For the six studies that were interventional, goal setting was the primary intervention activity, but goal attainment was only the primary outcome for three studies.

Studies were conducted in several different settings, including the hospital (*n* = 10), outpatient centers (*n* = 7), community (*n* = 7), home (*n* = 4), rehabilitation facilities (*n* = 2), and the emergency room (*n* = 1). The median age of the aging adults was 76 years (range: 56–86) and most samples were female (63%). Aging adults reported a variety of conditions including dementia, stroke, and other chronic conditions. The median age of the care partners was 64 years (range: 51–80).

### Narrative Synthesis

Our thematic analysis of included studies revealed three major themes that convey the scope of the research on this topic: types of goals, goal-setting process, and level of care partner involvement.

#### Types of goals

The types of goals in the included studies varied considerably. The most common goal that aging adults set was related to their specific medical conditions (*n* = 12). These included symptom management, medication management, and finding well-suited treatment plans (24–35). Many studies also revealed that aging adults wanted to remain at home (*n* = 10), maintain independence/autonomy (*n* = 9), and maintain or improve their functioning (*n* = 9) (24–27,29,31,32,36–45). Aging adults in these studies also wanted to maintain their general well-being and quality of life (*n* = 7), socialize (*n* = 8), pursue leisure activities (*n* = 5), meet their spiritual needs (*n* = 2) and be productive (*n* = 4) (24,26–29,31,35–37,43–48). Five studies reported on aging adults who set goals related to their planning for the future and end-of-life care (26–28,31,47,49). Some studies reported that aging adults had a goal of not being a burden for their care partner or family (*n* = 4) (24,32,45,47). A few studies (*n* = 3) had aging adults who reported goals related to having good relationships with their clinicians (24,31,37). One study reported on aging adults with financial goals (34).

Many care partners shared similar goals with the aging adults, however, their priorities differed. The most common goals for care partners were related to medical care (*n* = 8) (24–26,29,30,33–35). Keeping the aging adult at home was also a frequent goal for care partners (*n* = 6), however, independence (*n* = 4) and functional goals (*n* = 3) were not as common (25,26,29,31,34,39,45). Care partners also introduced a new goal related to aging adult safety (*n* = 4) (27,28,35,45). Care partners in these studies wanted the aging adult to maintain their general well-being and quality of life (*n* = 3), socialize (*n* = 5), pursue leisure activities (*n* = 2), meet their spiritual needs (*n* = 1), and be productive (*n* = 2) (24–26,28,29,34,35,41–43). Four studies reported on care partners who set goals related to planning for the future and end-of-life care for the aging adult (26,31,47,49). Five studies had care partners who reported goals related to having good relationships with the aging adult’s clinicians (24,26,27,31,37). Three studies reported on care partners with financial goals (24,27,34).

**Figure 2.**
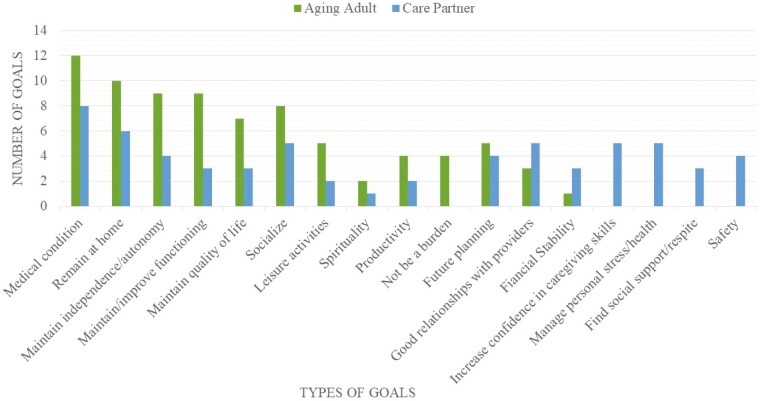
Frequency of reported goal types for care partners and aging adults. The number of each goal type reported is not weighted based on sample size and is only intended to show an overview of the common goal types for aging adults and their care partners. For example, goals related to a medical condition were reported 12 times by the aging adult across all included studies.

In addition to goals related to the aging adult, some studies reported on care partner-specific goals. Five studies revealed care partner goals related to increasing confidence in their caregiving abilities/support of aging adults (24,27,28,31,39). Five studies also identified care partner goals related to managing personal health and stress (27,28,31,32,40). Three studies described care partners who aimed to find social support and respite (27,28,32). One study found care partners who wanted to avoid intensive care tasks and improve dementia-related education for the community (27). Figure 2 shows the number of times different goals were reported by care partners and aging adults across all studies.

#### Goal-setting process

Another major theme was the goal-setting process, including the methods used to elicit goals, the barriers to goal setting, and the assessment of goal attainment. Several different measures were used to facilitate the goal-setting process. Nine studies used a quantitative measure, such as Goal Attainment Scaling (GAS) (50), the Life Priorities Survey (51), the Bangor Goal Setting Interview (52), or the Canadian Occupational Performance Measure (53). Most studies used an open-ended question to obtain goals (see Table 1 for summaries related to the goal-setting process; and the Supplementary Table, Section 3, for the full summary of the included articles). Some questions were more general, “What goals are important to you?” while others were slightly more targeted, “What are the important goals for your dementia care?”

**Table 1.** Summary of Included Articles

Author (Year), location	Name of measure or question related to goal-setting	Types of goals reported by aging adult	Types of goals reported by care partner	Goal attainment	Goal concordance (Y/N)
Almborg (2009), Sweden	Relative’s Questionnaire about Participation in Discharge planning (R-QPD) (54)	NR	NR	NR	N
Bogardus (1998), USA	“What are your goals for the patient’s/your care?”	Maintaining general wellbeing	Home safety, social and family relationships	NR	N
Bradley (1999), USA	“What are your goals for the patient’s/your care?”	Independence	Independence, dignity	NR	N
Bradley (2000), USA	“What are your goals for [patient’s name] care regarding his/her ability to do day-to-day activities?”	Home related, self-management, health promotion, health condition specific, home safety, socialization, economic stability, independence	Caregiver burden, home related, self-management, health promotion, health condition specific, home safety, socialization, economic stability, emotional stability, education/referrals	NR	N
Brock (2009), Australia	Goal attainment scaling (50)	Home related, self-management, health promotion, health condition specific, home safety, socialization, communication	Home related, self-management, health promotion, health condition specific, home safety, socialization, communication	20%	N
Clair (2021), USA	open-ended questions about what goals were important to participants followed by prompts about the importance of goals	Home related, self-management, health promotion, health condition specific, home safety, socialization, independence, end-of-life	Home related, self-management, health promotion, health condition specific, home safety, emotional stability, socialization, access to services and support, end-of-life wishes	NR	N
Clare (2010), UK	Canadian occupational performance measure (53)	Home related, self-management, health promotion, health condition specific, home safety, leisure	NR	full achieved: 46%; partially achieved: 50%; not achieved: 4%	N
Coeling (1999), USA	Open-ended question about goals	Independence	Independence	NR	N
Coleman (2015), USA	“What is one personal goal that is important for you to achieve in the next month?”	Self-management, health condition specific, socialization, function, employment/volunteer roles	Self-management, health condition specific, socialization, function, employment/volunteer roles	26 % Have met as well as expected; 26% Have met better than expected	Y
DeGroot (2021), USA	Not goals specifically, but asked about end-of-life and quality of life	somewhat related to end-of-life; not wanting to think about it vs. preparing; independence; enjoying everyday life; not being a burden;	somewhat related to end-of-life; not wanting to think about it vs. preparing; independence; enjoying everyday life; having resources	NR	N
Giosa (2022), Canada	Can you tell me about a time when you felt that health care providers treated you with respect, listened to your input, and acknowledged your choices? Can you tell me about a time when health care providers took the time to talk to you about your/ your family/friend’s needs and preferences for care?	Home related, self-management, health promotion, health condition, home safety	collaboration is easier when older adults and caregivers lead the way. doing “with” instead of doing “for” promotes participation relational communication involves two-way information sharing. seeing beyond age enables respect and dignity	NR	N
Giovannetti (2021), USA	Goal Attainment Scaling (50)	Home related, self-management, health promotion, health condition specific, home safety, economic stability, emotional stability, socialization	Home related, self-management, health promotion, health condition specific, home safety, socialization	74%	N
Glazier (2004), USA	Open-ended questions	Home related, self-management, heath condition, socialization, spiritual, future planning, dental care, memory	Home related, self-management, heath condition, socialization, spiritual, future planning, sexual functioning, communication with the team, and smoking	NR	Y
Heid (2016), USA	When there are multiple people involved in making decisions in daily life, we know that things can be difficult. We would like to hear more about how this works in your relationship. Describe one instance where you did see eye-to-eye with your RELATIVE?	Self-management, socialization		NR	N
Hindle (2018), UK	Bangor Goal‐Setting Interview (52)	Home related, self-management, health promotion, home safety, socialization	Caregiver burden, home related, self-management, health promotion, home safety, emotional stability/management	At the 6-month follow-up, there were main effects favoring CR for participants’ self-rated goal attainment on the BGSI (*F*(1,18) = 6.39, *p* = .008).	N
Jennings (2017), USA	“What are the important goals for your dementia care?”’ Caregivers were asked to answer the questions considering the person with dementia’s values and preferences	Self-management, health promotion, health condition specific, socialization, not be a burden, living at home	Caregiver burden, home safety, socialization, accessing services and supports, keeping older adult at home	NR	N
Jennings (2018), USA	Goal Attainment Scaling (50)	Health promotion, health condition specific, socialization, physical safety, living at home	Caregiver burden, minimize family conflict	74%	N
Kuluski (2013), Canada	NR, getting at patient goals from perspectives of patient, caregiver, and physician	Health promotion, health condition specific, preparation for future needs/more or different services	Health promotion, health condition specific, home safety, doing tasks for patient, preparation for future needs/more or different services	NR	Y
Lamas (2017), USA	What are your most important goals given your medical condition?	Self-management, health promotion, health condition specific, socialization, be at home, be mentally aware, have decisions respected, not a burden, independent, be spiritually and emotionally at peace, live as long as possible	Self-management, health promotion, health condition specific, socialization, be at home, be mentally aware, have decisions respected, not a burden, independent, be spiritually and emotionally at peace, live as long as possible	NR	N
Needham (2004), UK	NR, open-ended question about goals	Self-management, health condition specific	Self-management, health promotion, health condition specific	fully achieved: 37%, partially achieved: 42%, not achieved at all: 10%	Y
Phelps (2022), UK	Goals of care	Self-management, health promotion, socialization, be involved in decision-making	Self-management, health promotion, health condition specific, socialization	NR	N
Redfern (2002), UK	NR, open-ended question about goals	Home related, self-management, health promotion, health condition specific, home safety, socialization	Home related, self-management, health promotion	NR	N
Rockwood (2007), Canada	Goal Attainment Scaling (50)	Health condition specific	Health condition specific	58% in galantamine group; 24% in placebo group	Y
Shafir (2016), USA	NR, open-ended question about goals	Self-management, health promotion, health condition, comfort and remaining at home	Health condition specific	NR	N
Shaw (2020), UK	Goal Attainment Scaling (50)	Home related, self-management, health promotion, health condition specific, emotional stability/management, pain, communication	Supported patient goals	84%	N
Shin (2018), South Korea	asked about treatment goal (cure, life‐prolonging, and symptom relief)	Treatment goal	Treatment goal	NR	Y
Tsuda (2019), Japan	Asked about patient’s expressed wishes and surrogate’s preferred care goals for patient	End-of-life wishes	End-of-life wishes	NR	Y
Vluggen (2020), The Netherlands	Goal Attainment Scaling (50)	Self-management, other	Self-management, other goal for self	NR	N
Wilson (2014), USA	interview about “possible selves”: “what you would like to become or what you would like to happen in the future	Health promotion, health condition specific, economic stability	Self-management, health promotion, health condition specific, economic stability, emotional stability	NR	N
Wyman (2020), USA	“Tell me about some hopes or wishes you have for your health.	Self-management, health promotion, health condition, home safety, socialization, alleviating discomfort, autonomy and control, leaving a legacy, extending life security	Self-management, health promotion, health condition, economic stability, emotional stability, socialization, alleviating discomfort, autonomy and control, leaving a legacy, extending life security	NR	N
Zupa (2022), USA	collaborative goal-setting and action-planning	Health	Support aging adult goals.	NR	N

Participants also identified barriers that hindered the goal-setting process. One major barrier was the communication between clinicians, patients, and care partners. Participants in a study by Giosa et al. (39) revealed that aging adult participants found clinicians to be patronizing and reported that they made assumptions about patients’ hearing, sight, cognition, or care needs. In other cases, aging adults experienced communication challenges related to their own health conditions or treatments that complicated the goal-setting process (40,41,55,56). One study also reported that clinicians felt that goals set by care partners and aging adults could be unrealistic and frequently identified short evaluation times as a barrier to goal setting (40).

Another aspect of goal setting that was discussed in ten studies was goal attainment. In studies that reported goal attainment, the percentage of goals attained ranged from 20% to 84%. In some studies, goal attainment was classified as partially achieved or fully achieved, while others characterized goal attainment based on the measure used (eg, GAS). Several authors also examined factors contributing to goal attainment. Brock et al. (2009) found that lower levels of depression and higher self-efficacy were associated with goal attainment. Coleman et al. (2015) reported goal attainment was related to whether aging adults were living with their care partner and the type of goal set. In contrast, Jennings et al. (2018) and Giovannetti (2021) found that goal attainment was not related to goal type or difficulty.

#### Level of care partner involvement

Goal setting for aging adults was conducted with varying levels of care partner involvement. In some studies, care partners served in a supportive role, providing input when needed for the aging adults’ goals. Four studies described care partner’s helping or supporting the aging adults in the goal-setting process or “doing with” (29,44,57,58). Care partners were asked to support the goal-setting process led by the aging adult or were jointly setting goals with the aging adult. In the study by Clare et al. (2010) patients with probable Alzheimer’s disease were asked to set goals related to self-care, leisure, and productivity. Care partners in this study, when available, were asked to support the goal attainment process and progress checking during follow-up visits. In the remaining studies that employed this “doing with” approach, care partners and aging adults determined goals for the aging adult in a collaborative approach. In a study by Shaw et al. (2020) patients who had a stroke were asked to complete joint rehabilitation goal setting with their carer in collaboration with their care team. Similarly, in a study by Zupa et al. (2022) care partners, called “supporters,” were asked to complete a collaborative goal setting and action-planning scale with the aging adult. Almborg et al. (2009) studied the extent to which care partners were included in the goal-setting process as supporters, with 80% of participants reporting that they were not included in this process.

Most studies (*n* = 20) reported on care partners who participated equally in the goal-setting process. In these studies, participants were either asked to set goals in a non-specific manner, were specifically asked to set goals for the aging adult’s care plan, or were asked to set goals for themselves and the aging adult. These studies generally used an open-ended question (*n* = 17) to elicit goals from aging adults and their care partners. Many evaluations of goals were conducted with care partners and aging adults separately (*n* = 9), while some were conducted with them together (*n* = 5), and others were dependent upon participant preference (*n* = 6). Despite the inclusion of care partners as an independent informant in this level of involvement, they were inconsistently asked about self-directed goals and only a few studies reported on “do for self” goals created by the care partners (*n* = 8).

Half of the studies included in this equal care partner involvement category also reported on goal concordance between aging adults and their care partners. Four of these studies quantitatively assessed goal concordance with an average percent agreement between care partners and aging adults’ goals of 50.3% (25,26,47,59). The remaining studies included qualitative descriptions of goal concordance. Heid et al. (2016) reported that when there were differences between care goals for daughter care partners and their parents, it was more likely that the care partner would “reason” with the older adult and that the older adult would just “let go” of their goal. In the study by Jennings et al. (2017), one major conflict that was identified was the care partner’s goal of ensuring the safety of the aging adult and the older adult with dementia’s goal of continuing to live independently. Kuluski et al. (2013) found that disagreement in goals was more common when the aging adult had a decline in their functioning or cognitive abilities. In a study by Needham et al. (2004), researchers found that when goals differed between aging adults and their care partners, this could sometimes be attributed to care partners’ goals relating to additional issues that the aging adult faced but did not recognize.

A third level of involvement included care partners who set goals on behalf of the aging adult for whom they provided care or “doing for.” This level of involvement was more common when the aging adult experienced communication or cognitive impairments that hindered their ability to set independent goals. Aging adults in these studies experienced different conditions that made it more challenging to report goals. Lamas et al. (2017) conducted interviews with chronically, critically ill long-term acute care patients or their surrogates about goals or “life priorities.” In this study, the care partner was only involved if the aging adult could not complete the interview. In a few studies there was a mix of types of care partner involvement dependent upon aging adult preference or ability. Care partners in these studies were interviewed, alongside or separate from participants according to individual preferences (37,42). Similarly, Redfern et al. (2002) interviewed people with dementia and their care partners. Researchers noted that in general goal setting was driven by the care partners, however, in some cases the aging adult would voice their opinions.

## Discussion

The findings of this scoping review reveal that care partner inclusion in goal setting varies significantly with some care partners taking a supportive role for the aging adult, others collaborating in goal setting, and some leading the goal-setting process. Goal types also differed between care partners and aging adults. Studies reporting on goal concordance (*n* = 10) revealed varying alignment of goals within dyads. When goal discordance exists within a dyad, it is important to implement procedures that promote collaboration between aging adults, care partners, and clinicians during the goal-setting process. This type of approach is supported by the recent proposal from the Centers for Medicare & Medicaid Services (CMS) to engage care partners in treatment planning to support people with Medicare (60). Interestingly, we also found that studies reporting on goal setting for aging adults and care partners have increased over time with most articles in this review published after 2015. These findings may reflect recent health care trends and policies. More emphasis on goal setting could be attributed to person-centered and family-centered care practices, the Affordable Care Act, and the newly released proposed CMS payment rules (60,61).

The lack of a systematic method for including care partners in goal setting reveals a gap in current care practices for aging adults. This gap could be attributed to insufficient training of clinicians in effective communication practices for including care partners in health care decision-making. The current review identified several studies that described poor communication between patients, care partners, and clinicians as negatively affecting goal setting (39–41,55). Existing evidence also suggests that poor clinician communication can contribute to worse health outcomes and increased caregiver burden (62,63). Giosa et al. (2022) specifically commented on the negative effect that patronizing clinicians can have on the goal-setting process, contributing to worse life satisfaction and subjective health for aging adults. Other researchers have found that clinician ageism and paternalism contribute to inaccurate assumptions about aging adults’ preferences for their medical care and goals of care (10,64,65). Consequently, better goal-setting processes and increased care partner involvement in goal setting may hinge on improving clinician communication (66).

Another barrier to adequate care partner inclusion in clinical visits was limited time (40). Lack of time is frequently identified as a barrier to implementing recommended clinical practice guidelines (67). One method to address this barrier and facilitate the inclusion of care partners in a discussion of goals during clinical visits is to implement pre-visit assessments. Assessments that can be completed by aging adults and their care partners through electronic health records have been successful in other contexts and could prepare clinicians, care partners, and aging adults for goal setting during routine care visits (68). In addition, involving other health care professionals (ie, nonphysicians) like occupational therapists could facilitate collaborative goal setting (69).

The review also revealed that care partner goals for themselves were limited, potentially indicating a lack of recognition of care partners as important members of the care team who also need support to care for the aging adult. A study conducted by the National Alliance for Caregiving and the AARP revealed that only 29% of care partners were asked by a clinician about what they need to support an aging adult (15). The current review reported similar trends with many prompts to elicit goals focused on the aging adult or their treatment plan specifically, neglecting care partner needs. This problem is compounded by inconsistent use of measures to facilitate goal-setting and assess goal attainment which creates a barrier to routine evaluation of care partner and aging adult goals in clinical practice and research. We are unable to make reliable comparisons of the effectiveness of interventions that involve goal-setting when measures are heterogeneous. In this review, nine studies used a quantitative measure to assess goals, but most studies used an open-ended question to obtain goals. An objective measure could more easily indicate improvement and progress on the goals set. Although the GAS was used in some studies included in this review to describe goal attainment, there is currently no recommended way to evaluate dyadic goals using the GAS.

Studies in this review were conducted in a variety of settings, with most studies taking place in the hospital and home health settings and a few occurring in community-based settings, suggesting the use of goal setting as more reactive rather than preventative. Goal-setting was used to help aging adults after a health event (eg, stroke, hospitalization, and injury) rather than as part of a routine primary care visit to prevent negative health outcomes from occurring. The practice of goal-setting has been more routinely and effectively used in diabetes care (57,70), but less commonly used in general primary care. Including goal-setting in primary care and community-based services to help aging adults and their care partners establish healthy routines and prepare their homes for challenges that may come with aging should be prioritized. Interventions for people with dementia and their care partners like the Support, Health, Activities, Resources, and Education (SHARE), have tested effective methods to proactively create a care plan with the dyad, and similar approaches could be used for the general aging adult population (71). The current review revealed that remaining at home was a common goal for both members of the dyad, making a preventive approach that incorporates home adaptations critical (72,73).

This scoping review uncovered several gaps and directions for future research. First, there is a clear need for the development and implementation of a standardized process to facilitate collaborative goal-setting in routine care. This approach will likely need to address existing barriers in health care that hinder routine involvement of care partners like limited time in visits and insufficient communication skills (39–41,55,67). Second, we need to better understand how to encourage care partner goal-setting as well, aligning with recommendations from the 2022 National Strategy to Support Family Caregivers (74,75). Third, there is a need for a goal-setting and attainment scale that can be incorporated into dyadic or family-centered interventions. Fourth, the use of technology to facilitate goal-setting was rare in the studies in this review. With increasing efforts to enforce collaborative care planning in clinical practice, technology will likely play a pivotal role in promoting goal-setting (76). Future research is needed to examine best practices for incorporating dyadic goal-setting using technology. Fifth, this review uncovered an increase in goal-setting focused studies in the last 10 years. Future research exploring connections between goal-setting activity changes and policy, such as an environmental policy scan, could improve our understanding of this trend. Last, we need to examine goal-setting across more diverse populations and with different care partner/aging adult relationship types to uncover cultural and relationship differences in this process (40,77). Although the current review included studies that were set in many different countries, the study designs were heterogeneous, making comparison across studies challenging. Further exploration of cultural impacts on goal-setting in care partner and aging adults’ relationships is needed.

### Limitations

Although this review provides an overview of the understudied topic of goal-setting for aging adults and care partners, there are important limitations to consider. First, only studies that were available in English were included. Additionally, we did not include any “gray” literature, instead focusing on the peer-reviewed literature. Last, we did not perform any quality appraisal of the included studies. Although this practice is common for scoping reviews, the evidence presented should be considered with this limitation.

## Conclusion

We conducted a scoping review to better understand how goal-setting occurs between aging adults and their care partners. We found that aging adults and their care partners shared several similar goals, however they also had unique goals, sometimes competing goals, that were important to consider for care planning. The level of care partner involvement in goal-setting is highly variable and future research should aim to identify methods to increase routine involvement of care partners in goal-setting as care partners have the power to encourage goal progress after the clinician visit. The review uncovered several areas for future research and practice.

## Supplementary Material

igad135_suppl_Supplementary_Material
