# Supplementary material for: Goal Setting for Aging Adults and Care Partners: A Scoping Review
Source: Innov Aging. 2023 Dec 22;8(1):igad135. doi: 10.1093/geroni/igad135 (PMC10838149; doi:10.1093/geroni/igad135)
Supplement: igad135_suppl_Supplementary_Material [file igad135_suppl_supplementary_material.docx]

**Online Supplementary Material**

**Section 1. Search Strategy**

***PubMed***

(Elder*[tiab] OR aged[tiab] OR "older adults" OR geriatric OR "senior citizens" OR "Aged"[Mesh] OR patient*[tiab])

AND

("Caregivers"[Mesh] OR "Family"[Mesh] OR Caregiver[tiab] OR Caregiving[tiab] OR Carer*[tiab]) AND

((Goal-setting OR "goal setting" OR "attain* goal*" OR Goals[Mesh]))

AND

(community OR at-home OR "at home" OR home[tiab] OR discharge[tiab] OR “primary care”[tiab])

Filters: English = 185 results

***PsycINFO***

goal setting or goal-setting or goal planning or goal attainment or setting goals OR goals

AND ( (DE "Geriatric Patients" OR DE "Older Adulthood" ) OR TI ( elder or older or elderly or aged or geriatric or senior OR aged ) OR AB ( elder or older or elderly or aged or geriatric or senior OR aged )

AND

caregiv*

Academic Journals in English = 402 results

***Socindex***

Search 1

( ( ((DE "GOAL (Psychology)") OR TI ( "goal set*" OR "setting goal*" OR aspirations OR motivation OR goal* ) OR AB ( "goal set*" OR "setting goal*" OR aspirations or motivation OR goal*)

AND (DE "OLDER people") OR (DE "OLD age") OR TI (elderly or older or geriatric OR aged OR senior ) OR AB ( elderly or older or geriatric OR aged OR senior )

AND

TI ( caregiver* or caregiving OR carers ) OR AB ( caregiver* or caregiving OR carers )

Academic Journals in English = 200 results

Search #2

goals and goal setting

AND

eldery or geriatric of older adult or aged elderly or aged or older or elder or geriatric

AND caregiv*

Academic Journals in English = 58 results. Final 228 GOAL setting (Psychology) Use GOAL (Psychology)

***CINAHL***

( (MH "Frail Elderly") OR (MH "Aged") OR (MH "Aged, 80 and Over") OR (MH "Geriatrics") ) OR TI ( elderly or aged or older or elder or geriatric or older adults OR senior ) OR AB ( elderly or aged or older or elder or geriatric or older adults OR senior )

AND ( (MH "Goal-Setting") OR (MH "Goal Attainment") OR (MH "Motivation") ) OR TI ( goal setting or goal-setting or goal planning or goal attainment OR motivation ) OR AB ( goal setting or goal-setting or goal planning or goal attainment OR motivation )

AND (MH "Caregivers") OR TI ( caregiving OR caregiver OR carer ) OR AB ( caregiving OR caregiver OR carer )

Academic Journals in English, Aged 65+, Aged, 80 & over = 612 results.

**Section 2. Data Extraction Form**

| **Data Element** | **Response Options** |
| --- | --- |
| *General Study Characteristics* | |
| First author’s last name | Open-ended |
| Publication year | Open-ended |
| Study type | Intervention, observational, longitudinal |
| Study methodology | Quantitative, qualitative, mixed methods |
| Study location (country) | Open-ended |
| Study setting | Emergency department, hospital, home-based, nursing home, assisted living, community-based (e.g., senior center), rehabilitation facility, hospice, outpatient center, other, not reported |
| *Characteristics of Aging Adults* | |
| Age of aging adults | Open-ended |
| Gender of aging adults | Open-ended |
| Race of aging adults | Open-ended |
| Health condition (e.g., dementia, stroke, heart failure, healthy) | Open-ended |
| Education level | Open-ended |
| Disease duration | Open-ended |
| Income level | Open-ended |
| Employment status | Full-time, part-time, not employed, retired, other, not reported |
| Area of occupation | Open-ended |
| *Characteristics of Care Partners* | |
| Age of care partners | Open-ended |
| Gender of care partners | Open-ended |
| Health condition (e.g., dementia, stroke, heart failure, healthy) | Open-ended |
| Education level | Open-ended |
| Duration of care | Open-ended |
| Income level | Open-ended |
| Employment status | Full-time, part-time, not employed, retired, other, not reported |
| Area of occupation | Open-ended |
| Caregiving intensity (hours/week or hours/day) | Open-ended |
| Relationship types (care partner and aging adult relationship) | Spouse/partner, child (son, daughter, step-son, step-daughter, daughter-in-law, son-in-law), friend, neighbor, sibling, other relative (e.g., cousin, uncle, aunt) |
| Are care partner and aging adult cohabitating? | Open-ended |
| Mention of multiple care partners? | Open-ended |
| *Goal Setting Content* | |
| Type of goal setting assessment | Standardized measure (e.g., Goal Attainment Scale, Canadian Occupational Performance), Non-standardized measure (e.g. survey, free response during an interview) |
| Delivery mode (aging adult) | Self-report, by phone, in person, other |
| Delivery mode (care partner) | Self-report, by phone, in person, other |
| Name of goal setting measure or question in interview or survey related to goal setting | Open-ended |
| Goal setting for aging adult and care partner | Together, separate, other |
| Frequency of goal setting (e.g., once at the beginning of study, multiple times during study) | Open-ended |
| *Report of Goals* | |
| Types of goals reported by aging adult | Home-related (e.g., laundry, cleaning), self-management (e.g., personal care, hygiene), health promotion (e.g., eating healthier, exercising), health condition specific (e.g., medication adherence), home safety, socialization, other |
| If other goals selected, list type of goals. | Open-ended |
| Types of goals reported by care partner | Caregiver burden/strain, home-related (e.g., laundry, cleaning), self-management (e.g., personal care, hygiene), health promotion (e.g., eating healthier, exercising), health condition specific (e.g. medication adherence), home safety, economic stability, emotional stability/management, socialization, other, other goals for self (as a care partner) |
| If other goals selected, list type of goals. | Open-ended |
| Goals achieved (% yes) – aging adult | Open-ended |
| Goals achieved (% yes) – care partner | Open-ended |
| *Other notes/relevant information* | |
| Any other information that might be good to include | Open-ended |
| Limitations listed | Sample size, study design, selection bias, measurement bias, other limitations |
| Other limitations listed | Open-ended |

**Section 3. Summary of Included Articles**

| **Author (Year), Location** | **Study Design/**  **Approach** | **Setting** | **Aging Adult Health Condition** | **Personnel Type** | **Name of measure or question related to goal setting** | **Types of goals reported by aging adult** | **Types of goals reported by care partner** | **Goal attainment** | **Goal concordance (Y/N)** |
| --- | --- | --- | --- | --- | --- | --- | --- | --- | --- |
| Almborg (2009), Sweden | Observational, Quantitative | Hospital | Stroke | NR | Relative’s Questionnaire about Participation in Discharge planning (R-QPD) ^77^ | NR | NR | NR | N |
| Bogardus (1998), USA | Observational, Qualitative | Outpatient Center | Dementia | Researcher | “What are your goals for the patient’s/ your care?” | Maintaining general wellbeing | Home safety, social and family relationships, | NR | N |
| Bradley (1999), USA | Observational, Qualitative | Hospital | NR | Researcher | “What are your goals for the patient's/your care?” | Independence | Independence, dignity | NR | N |
| Bradley (2000), USA | Observational, Mixed Methods | Outpatient Center | Varying degree of cognitive impairment | Physicians and care managers | “What are your goals for [patient’s name] care regarding his/her ability to do day-to-day activities?” | Home related, self-management, health promotion, health condition specific, home safety, socialization, economic stability, independence | Caregiver burden, home related, self-management, health promotion, health condition specific, home safety, socialization, economic stability, emotional stability, education/referrals | NR | N |
| Brock (2009), Australia | Observational, Quantitative | Hospital | Acute stroke | Therapists | Goal Attainment Scaling^49^ | Home related, self-management, health promotion, health condition specific, home safety, socialization, communication | Home related, self-management, health promotion, health condition specific, home safety, socialization, communication | 20% | N |
| Clair (2021), USA | Observational, Qualitative | Community-based | Older adults with functional limitations | Researcher | open-ended questions about what goals were important to participants followed by prompts about the importance of goals | Home related, self-management, health promotion, health condition specific, home safety, socialization, independence, end-of-life | Home related, self-management, health promotion, health condition specific, home safety, emotional stability, socialization, access to services and support, end-of-life wishes | NR | N |
| Clare (2010), UK | Intervention for goal-oriented cognitive rehabilitation, Quantitative | Outpatient Center | Probable Alzheimer’s Disease | Researcher | Canadian Occupational Performance Measure^52^ | Home related, self-management, health promotion, health condition specific, home safety, leisure | NR | full achieved: 46%; partially achieved: 50 %; not achieved: 4% | N |
| Coeling (1999), USA | Observational, Qualitative | Home-based | Dependency level high | Researcher | Open-ended question about goals | Independence | Independence | NR | N |
| Coleman (2015), USA | Longitudinal, Quantitative | Hospital | participants admitted to the cardiovascular unit, the general medical-surgical unit, and the orthopedics unit. | Researcher | “What is one personal goal that is important for you to achieve in the next month?” | Self-management, health condition specific, socialization, function, employment/volunteer roles | Self-management, health condition specific, socialization, function, employment/volunteer roles | 26 % Have met as well as expected 26% Have met better than expected | Y |
| DeGroot (2021), USA | Observational, Qualitative | Hospital | Someone with a ventricular assist device | Researcher | Not goals specifically, but asked about end of life and quality of life | somewhat related to end of life; not wanting to think about it vs. preparing; independence; enjoying everyday life; not being a burden; | somewhat related to end of life; not wanting to think about it vs. preparing; independence; enjoying everyday life; having resources | NR | N |
| Giosa (2022), Canada | Observational, Qualitative | Community-based | Older adults receiving home care services for a variety of complex health issues including stroke, multiple sclerosis, amyotrophic lateral sclerosis, muscular dystrophy, Parkinson's disease, cancer, and dementia. | Researcher | Can you tell me about a time when you felt that healthcare providers treated you with respect, listened to your input, and acknowledged your choices? Can you tell me about a time when healthcare providers took the time to talk to you about your/ your family/friend's needs and preferences for care? | Home related, self-management, health promotion, health condition, home safety | collaboration is easier when older adults and caregivers lead the way. doing ‘with’ instead of doing ‘for’ promotes participation relational communication involves two-way information sharing. seeing beyond age enables respect and dignity | NR | N |
| Giovannetti (2021), USA | Observational, Quantitative | Outpatient Center | 50 and older with ADL or IADL impairment | Clinicians | Goal Attainment Scaling ^49^ | Home related, self-management, health promotion, health condition specific, home safety, economic stability, emotional stability, socialization | Home related, self-management, health promotion, health condition specific, home safety, socialization | 74% | N |
| Glazier (2004), USA | Observational, Qualitative | Hospital | Older adults with complex medical and psychosocial problems | Social workers | Open-ended questions | Home related, self-management, heath condition, socialization, spiritual, future planning, dental care, memory, | Home related, self-management, heath condition, socialization, spiritual, future planning, sexual functioning, communication with the team, and smoking | NR | Y |
| Heid (2016), USA | Observational, Qualitative | Community-based | Older adult | Researcher | When there are multiple people involved in making decisions in daily life, we know that things can be difficult. We would like to hear more about how this works in your relationship. Describe one instance where you did see eye-to-eye with your RELATIVE] | Self-management, socialization |  | NR | N |
| Hindle (2017), UK | Intervention for goal-oriented cognitive rehabilitation in Parkinson’s disease, Quantitative | Rehabilitation Facility | Parkinson's disease dementia/ DLB | Researcher | Bangor Goal‐Setting Interview ^51^ | Home related, self-management, health promotion, home safety, socialization | Caregiver burden, home related, self-management, health promotion, home safety, emotional stability/management | At the 6-month follow-up, there were main effects favoring CR for participants' self-rated goal attainment on the BGSI(F(1,18) = 6.39,P= 0.008). | N |
| Jennings (2017), USA | Observational, Qualitative | Community-based | Early stage dementia | Researcher | ‘What are the important goals for your dementia care?’’ Caregivers were asked to answer the questions considering the person with dementia’s values and preferences | Self-management, health promotion, health condition specific, socialization, not be a burden, living at home | Caregiver burden, home safety, socialization, accessing services and supports, keeping older adult at home | NR | N |
| Jennings (2018), USA | Observational, Mixed Methods | Outpatient Center | Dementia | Dementia care manager | Goal Attainment Scaling ^49^ | Health promotion, health condition specific, socialization, physical safety, living at home | Caregiver burden, minimize family conflict | 74% | N |
| Kuluski (2013), Canada | Observational, Mixed Methods | Outpatient Center | Two or more chronic conditions | Researcher | NR, getting at patient goals from perspectives of patient, caregiver, and physician | Health promotion, health condition specific, preparation for future needs/more or different services | Health promotion, health condition specific, home safety, doing tasks for patient, preparation for future needs/more or different services | NR | Y |
| Lamas (2017), USA | Observational, Mixed Methods | Hospital | respiratory failure 26%; surgical 26%; neurologic insult 16%; trauma 16%; cardiac disease 12%; neuromuscular 4% | Researcher (with input from clinicians in critical care and palliative care) | What are your most important goals given your medical condition? | Self-management, health promotion, health condition specific, socialization, be at home, be mentally aware, have decisions respected, not a burden, independent, be spiritually and emotionally at peace, live as long as possible | Self-management, health promotion, health condition specific, socialization, be at home, be mentally aware, have decisions respected, not a burden, independent, be spiritually and emotionally at peace, live as long as possible | NR | N |
| Needham (2004), UK | Observational, Qualitative | Hospital | Life threatening condition | Hospice Staff | NR, open-ended question about goals | Self-management, health condition specific | Self-management, health promotion, health condition specific | fully achieved: 37%, partially achieved: 42%, not achieved at all: 10% | Y |
| Phelps (2022), UK | Observational, Qualitative | Emergency Department | ED patient | Researcher | Goals of care | Self-management, health promotion, socialization, be involved in decision-making | Self-management, health promotion, health condition specific, socialization, | NR | N |
| Redfern (2002), UK | Observational, Qualitative | Community-based | Dementia | Researcher | NR, open-ended question about goals | Home related, self-management, health promotion, health condition specific, home safety, socialization | Home related, self-management, health promotion | NR | N |
| Rockwood (2007), Canada | Intervention to reduce verbal repetition, Quantitative | Community-based | Alzheimer’s disease | Clinician | Goal Attainment Scaling ^49^ | Health condition specific | Health condition specific | 58% in galantamine group; 24% in placebo group | Y |
| Shafir (2016), USA | Observational, Qualitative | Home-based | Homebound older adults | Researcher | NR, open-ended question about goals | Self-management, health promotion, health condition, comfort and remaining at home | Health condition specific | NR | N |
| Shaw (2020), UK | Intervention with rehabilitation goals post-stroke, Quantitative | Hospital | Stroke | Clinician | Goal Attainment Scaling ^49^ | Home related, self-management, health promotion, health condition specific, emotional stability/management, pain, communication | Supported patient goals | 84% | N |
| Shin (2018), South Korea | Observational, Quantitative | Hospital | Cancer | Researcher | asked about treatment goal (cure, life‐prolonging, and symptom relief) | Treatment goal | Treatment goal | NR | Y |
| Tsuda (2019), Japan | Longitudinal, Quantitative | Home-based | 75.4% of the patients were partially or totally ADL dependent, and 37.7% had severely impaired cognitive function | Researcher | Asked about patient’s expressed wishes and surrogate’s preferred care goals for patient | End-of-life wishes | End-of-life wishes | NR | Y |
| Vluggen (2020), Netherlands | Intervention with rehabilitation goals post-stroke, Mixed Methods | Rehabilitation Facility | Stroke | Care professional | Goal Attainment Scaling ^49^ | Self-management, other | Self-management, other goal for self | NR | N |
| Wilson (2014), USA | Observational, Mixed Methods | Home-based | Men with localized prostate cancer | Researcher | interview about "possible selves": “what you would like to become or what you would like to happen in the future | Health promotion, health condition specific, economic stability | Self-management, health promotion, health condition specific, economic stability, emotional stability | NR | N |
| Wyman (2020), USA | Observational, Qualitative | Community-based | High-need, high cost patients (multiple chronic conditions) | Geriatrician | “Tell me about some hopes or wishes you have for your health. | Self-management, health promotion, health condition, home safety, socialization, alleviating discomfort, autonomy and control, leaving a legacy, extending life security | Self-management, health promotion, health condition, economic stability, emotional stability, socialization, alleviating discomfort, autonomy and control, leaving a legacy, extending life security | NR | N |
| Zupa (2022), USA | Intervention for diabetes management, Quantitative | Outpatient center | Type 2 Diabetes | Health Coach | collaborative goal-setting and action-planning | health | Support aging adult goals. | NR | N |

**Section 4. Preferred Reporting Items for Systematic reviews and Meta-Analyses extension for Scoping Reviews (PRISMA-ScR) Checklist**

| **SECTION** | **ITEM** | **PRISMA-ScR CHECKLIST ITEM** | **REPORTED ON PAGE #** |
| --- | --- | --- | --- |
| **TITLE** | | | |
| Title | 1 | Identify the report as a scoping review. | 1 |
| **ABSTRACT** | | | |
| Structured summary | 2 | Provide a structured summary that includes (as applicable): background, objectives, eligibility criteria, sources of evidence, charting methods, results, and conclusions that relate to the review questions and objectives. | 2 |
| **INTRODUCTION** | | | |
| Rationale | 3 | Describe the rationale for the review in the context of what is already known. Explain why the review questions/objectives lend themselves to a scoping review approach. | 5 |
| Objectives | 4 | Provide an explicit statement of the questions and objectives being addressed with reference to their key elements (e.g., population or participants, concepts, and context) or other relevant key elements used to conceptualize the review questions and/or objectives. | 5 |
| **METHODS** | | | |
| Protocol and registration | 5 | Indicate whether a review protocol exists; state if and where it can be accessed (e.g., a Web address); and if available, provide registration information, including the registration number. | N/A |
| Eligibility criteria | 6 | Specify characteristics of the sources of evidence used as eligibility criteria (e.g., years considered, language, and publication status), and provide a rationale. | 6 |
| Information sources* | 7 | Describe all information sources in the search (e.g., databases with dates of coverage and contact with authors to identify additional sources), as well as the date the most recent search was executed. | 5 |
| Search | 8 | Present the full electronic search strategy for at least 1 database, including any limits used, such that it could be repeated. | 5 |
| Selection of sources of evidence† | 9 | State the process for selecting sources of evidence (i.e., screening and eligibility) included in the scoping review. | 6 |
| Data charting process‡ | 10 | Describe the methods of charting data from the included sources of evidence (e.g., calibrated forms or forms that have been tested by the team before their use, and whether data charting was done independently or in duplicate) and any processes for obtaining and confirming data from investigators. | 7 |
| Data items | 11 | List and define all variables for which data were sought and any assumptions and simplifications made. | 7 |
| Critical appraisal of individual sources of evidence§ | 12 | If done, provide a rationale for conducting a critical appraisal of included sources of evidence; describe the methods used and how this information was used in any data synthesis (if appropriate). | N/A |
| Synthesis of results | 13 | Describe the methods of handling and summarizing the data that were charted. | 7 |
| **RESULTS** | | | |
| Selection of sources of evidence | 14 | Give numbers of sources of evidence screened, assessed for eligibility, and included in the review, with reasons for exclusions at each stage, ideally using a flow diagram. | 26 |
| Characteristics of sources of evidence | 15 | For each source of evidence, present characteristics for which data were charted and provide the citations. | 28 |
| Critical appraisal within sources of evidence | 16 | If done, present data on critical appraisal of included sources of evidence (see item 12). | N/A |
| Results of individual sources of evidence | 17 | For each included source of evidence, present the relevant data that were charted that relate to the review questions and objectives. | 28 |
| Synthesis of results | 18 | Summarize and/or present the charting results as they relate to the review questions and objectives. | 7 |
| **DISCUSSION** | | | |
| Summary of evidence | 19 | Summarize the main results (including an overview of concepts, themes, and types of evidence available), link to the review questions and objectives, and consider the relevance to key groups. | 14 |
| Limitations | 20 | Discuss the limitations of the scoping review process. | 18 |
| Conclusions | 21 | Provide a general interpretation of the results with respect to the review questions and objectives, as well as potential implications and/or next steps. | 18 |
| **FUNDING** | | | |
| Funding | 22 | Describe sources of funding for the included sources of evidence, as well as sources of funding for the scoping review. Describe the role of the funders of the scoping review. | 20 |

JBI = Joanna Briggs Institute; PRISMA-ScR = Preferred Reporting Items for Systematic reviews and Meta-Analyses extension for Scoping Reviews.

* Where *sources of evidence* (see second footnote) are compiled from, such as bibliographic databases, social media platforms, and Web sites.

† A more inclusive/heterogeneous term used to account for the different types of evidence or data sources (e.g., quantitative and/or qualitative research, expert opinion, and policy documents) that may be eligible in a scoping review as opposed to only studies. This is not to be confused with *information sources* (see first footnote).

‡ The frameworks by Arksey and O’Malley (6) and Levac and colleagues (7) and the JBI guidance (4, 5) refer to the process of data extraction in a scoping review as data charting*.*

§ The process of systematically examining research evidence to assess its validity, results, and relevance before using it to inform a decision. This term is used for items 12 and 19 instead of "risk of bias" (which is more applicable to systematic reviews of interventions) to include and acknowledge the various sources of evidence that may be used in a scoping review (e.g., quantitative and/or qualitative research, expert opinion, and policy document).

*From:* Tricco AC, Lillie E, Zarin W, O'Brien KK, Colquhoun H, Levac D, et al. PRISMA Extension for Scoping Reviews (PRISMAScR): Checklist and Explanation. Ann Intern Med. 2018;169:467–473. [doi: 10.7326/M18-0850](http://annals.org/aim/fullarticle/2700389/prisma-extension-scoping-reviews-prisma-scr-checklist-explanation).
